# The Public School-Boy's Body

**Published:** 1892-10-01

**Authors:** 


### The Hospital

An Institutional Journal of

Science, Medicine, Nursing, and Philanthropy.

*For Hospitals, Asylums, Infirmaries, Dispensaries, Medical Practitioners, Students, Nurses, and the Charitable Public.*

Vol. XIII.—No. 314.] [Oct. 1, 1892.

### The Public School-boy's Body.

The mind of the Public School-boy may be superior to his body, but it must be admitted that his body is more in evidence. It has always been recognised that English Public Schools do not primarily make scholars; they make men first, and generally; they only make scholars now and again. The scholar, indeed, has ordinarily much to do with his own making: he is for the most part like the poet, *nascitur non fit.*

It is beginning to be recognised that the body is to a very marked extent a true index of the mind. The open countenance, the wholesome colour and form, the healthy skin, the clean limbs, the well-poised head, the swinging walk, and the honest laugh usually proclaim a boy of manly courage, of sweet and wholesome mind, and of practical and effective intellectual capacity: such a boy as would make a good parson, a worthy doctor, a useful engineer, a brave soldier, and an honourable Englishman in any capacity. On the other hand the ill-formed, thin, peevish, half-starved looking youth, although he may in some rare instances be amiable, patient, and brave, is much oftener twin in mind to his own ill-made and unfortunate body. Now and again he is exceptionally clever, a Rashleigh Osbaldestone, it may be, as cunning and unprincipled as he is clever. But even cleverness, Professor Lombroso notwithstanding, is not the common mark of the ill-formed boy: it is the exception. Taking the average youth of these two classes, it is abundantly evident that the sound mind usually corresponds with the sound body, and that physiological wholesomeness is the ordinary concomitant of mental capacity and moral worth.

If this reasoning be sound it is evident that our public schools have incurred great responsibilities in taking charge of the bodies of boys, as well as of their minds, at the very period of life when development is manifesting its most strenuous activity. How are they fulfilling these responsibilities? In the case of several schools it is said they are not fulfilling them well. Experienced medical men and trained physiologists have uttered notes of warning, which demand the most earnest and intelligent attention. The following, for instance, is said to be a sample of the day's dietary at one of our public schools. Breakfast is at a quarter past eight, and consists of bread and butter, with tea or coffee; dinner is at half-past one, and, of course, is the usual plain dinner of meat and pudding; tea is at seven, and the only solid fare supplied is, as at breakfast, bread and butter.

Now, if the authorities at our public schools knew as much of practical science as they know little, a dietary of this kind would be nothing short of disgraceful. We do not wish to speak strongly, but it is imperative to speak with such a degree of energy as will command the serious attention of those concerned. The first objection we have to the dietary is to its poverty; the second to its distribution. It is to all intents and purposes a diet which is but little superior to the prisoner's fare of bread and water. There is meat only once a day—and that for young, growing Englishmen of the better class. For our part—and we speak with careful sobriety—we do not think that any English boy can be properly fed unless he is supplied with a reasonable quantity of nitrogenous food three times a day. At breakfast there should be meat, or fish, or eggs; at dinner there should be meat of several kinds, as well as a various abundance of vegetables and puddings. But, since the dinner is served in the middle of the day, and there is much mental and physical work to be performed in the afternoon and evening, there should be eggs or fish provided for one of the evening meals.

With regard to distribution: It is manifest to every physiologist that the intervals between the meals are dangerously long. A dog will thrive on one meal a day, if it be a good one. But then it must be remembered that the dog is by nature a scavenger, and is picking up stray tit-bits all the day long. Moreover, the dog is a hoarder, and can always fly to his buried store of bones when hunger pinches. It need not be doubted either that men and women, under certain circumstances, can thrive and prosper on two meals a day. But then those men and women have different stomachs, different brains, and different habits from the public school-boy. A savage may gorge several pounds of half-cooked flesh, and may require no more for twenty-four hours. But what amount and quality of brain work could he do during the first twelve hours after his feast? He does not attempt any brain work at all; he goes to sleep, and sleeps off his debauch; for such feeding is physical debauchery.

The problem which the public schools have to solve is twofold: it is the rational feeding of brain workers, and the building up of muscular and healthy men. Now, we appeal to all brain workers to consider whether it is so much as possible to work well soon after a heavy meal! Every brain worker earlier or later finds out two things about his meals: he discovers that they must be reasonably frequent, and also that they must be very nutritious and sustaining. At the paiticular school which we have cited breakfast is served at a quarter-past eight and dinner at half-past one. That is an interval of five and a-quarter hours between the two meals. But the only solid food which is provided for breakfast is bread and butter. Every experienced person knows that in a case of this kind one of two things must happen: if the schoolboy is to avoid suffering the pangs of gnawing hunger and the dragging weariness of brain and limb which this hunger entails for two full hours before the time for dinner, he must stuff in a quantity of bread at breakfast which will be simply enormous, so enormous indeed as to make brain work almost impossible. If, on the other hand, he does not dispose of a quartern loaf at breakfast—and very few schoolboys are either able or willing to accomplish such a doughy feat—then he must be prepared to be hungry, angry, quarrelsome, and unfit for work for the two hours between half-past eleven and half-past one every day.

Similarly, the interval between dinner and tea, which is five and a-half hours, is a great deal too long; though, of course, the dinner requires more digestion than the breakfast, and is much more sustaining. But the present writer, if he were again a school-boy, would quarrel most of all with the arrangement which gives a miserable bread and butter meal at seven p.m., and an equally un-English bread and butter meal on the following day at a quarter-past eight a.m., with a fasting interval of thirteen and a-quarter hours between. There would be at least one public school Hampden if the writer were a boy again under those conditions. But space warns us to sum up. In a word, public school-boys should have four satisfying and nutritious meals a day. At one of them there should be abundance of meat; at two others there should be a reasonable quantity of nitrogenous food, such as meat, fish, or eggs. The English-born and English-bred youth of the public school class has inherited the need for this kind of dietary from a long line of robustly-fed ancestors. He cannot be developed to his best and strongest, either in body or in mind, without it.

